# Supplementary material for: The mechanism of glycosphingolipid degradation revealed by a GALC-SapA complex structure
Source: Nat Commun. 2018 Jan 11;9:151. doi: 10.1038/s41467-017-02361-y (PMC5764952; doi:10.1038/s41467-017-02361-y)
Supplement: Supplementary file 1 — Supplementary Information [file 41467_2017_2361_MOESM1_ESM.pdf]

Supplementary Figure 1

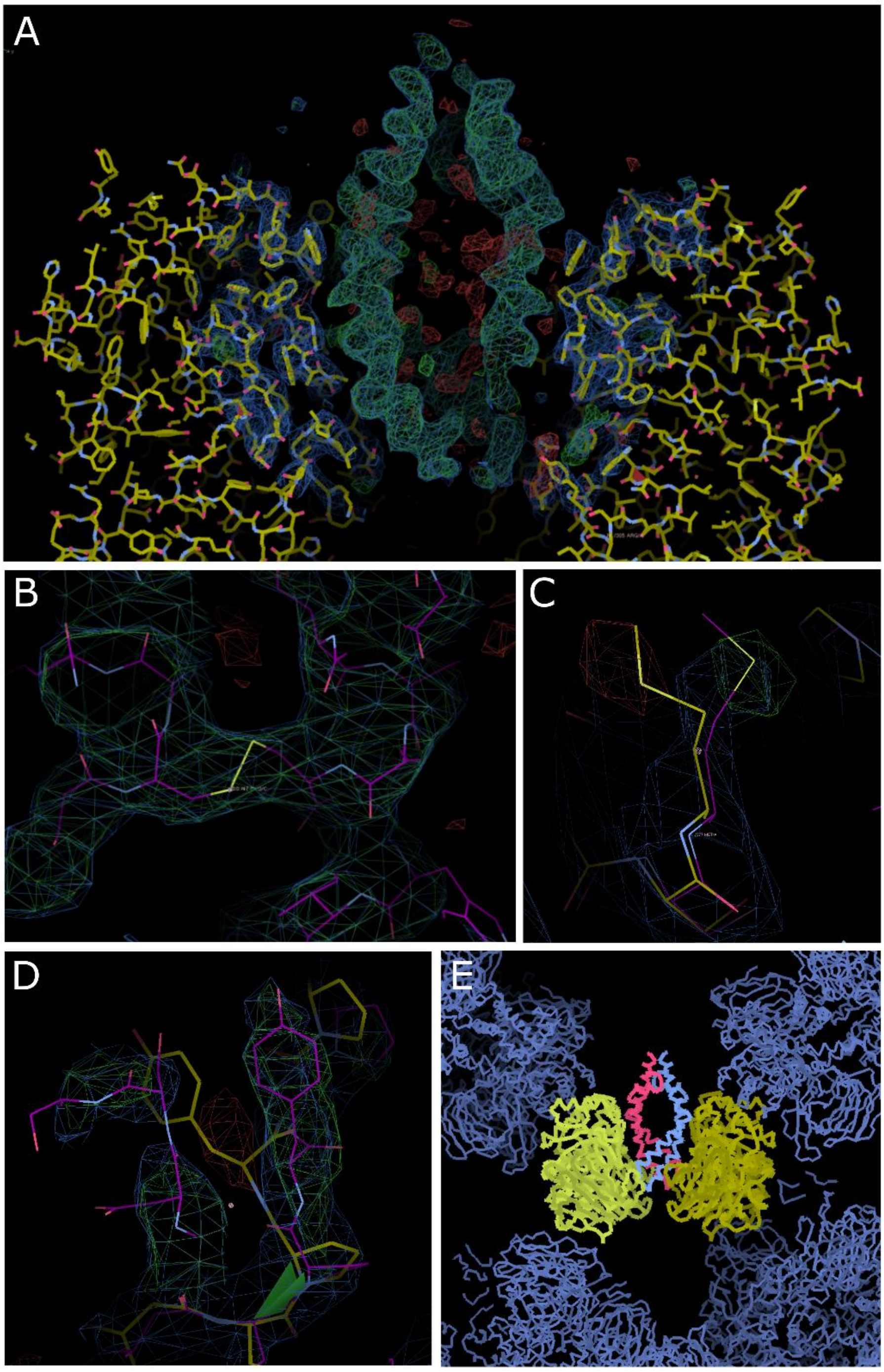

Supplementary Figure 1. Evidence for SapA positioning and sidechain modelling. Electron density maps calculated after molecular replacement with two molecules of GALC (PDB ID 4CCE [1]) but before any manual rebuilding are shown: $2F_o$-F_c, blue at 0.12 $e^-/Å^3$; and F_o-F_c, green/red for positive/negative at 3.0 σ. (A) Unbiased F_O-F_C difference density allows for clear identification and placement of SapA. (B) Disulfide bridges (three per SapA molecule) are clearly identifiable in unbiased F_o-F_c difference density, and allowed for unambiguous determination of the orientation and sequence register of the SapA molecule. The final refined SapA molecule (carbon atoms purple) is shown. (C and D) Examples of local remodelling of GALC, guided by F_O-F_C difference maps. The initial GALC model used for molecular replacement (carbon atoms yellow) and the final refined model (carbon atoms purple) are shown. (E) COOT screenshot illustrating the symmetry related molecules in the crystal lattice. For clarity, all chains are shown as backbone traces.

Supplementary Figure 2

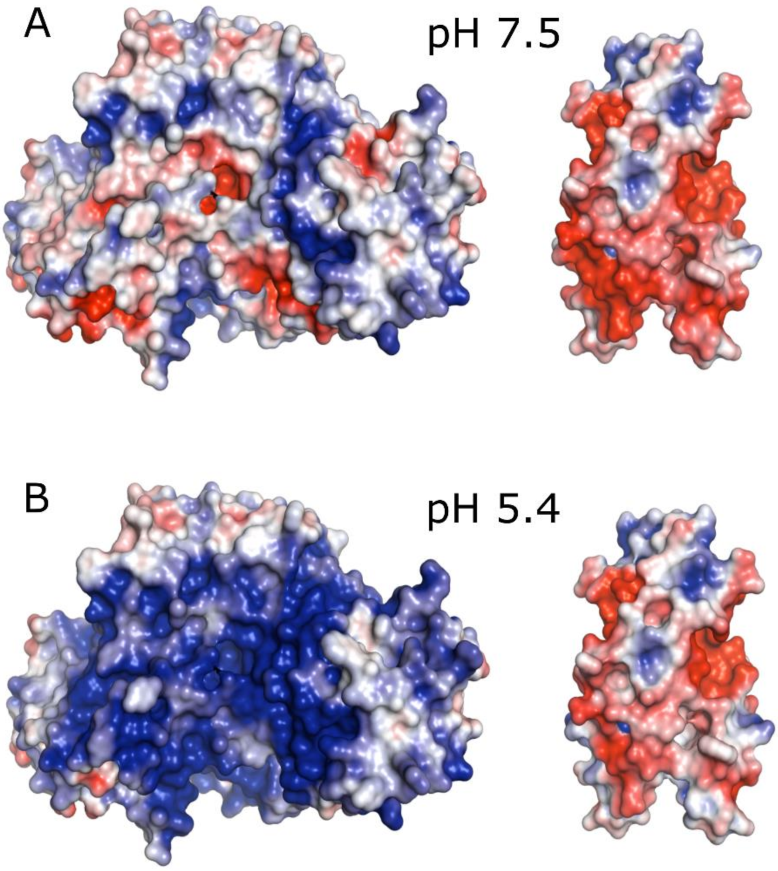

Supplementary Figure 2. Electrostatic surface potential of GALC and SapA at (A) pH 7.5 and (B) pH 5.4. The overall charge of GALC changes from -5 e^- to +15 e^- between pH 7.5 and pH 5.4, while SapA retains an overall negative charge at both pHs, -16 e^- to -10 e^-. This change in surface charge for GALC is particularly prevalent across the interaction interface with SapA. Thus, the transition in overall surface charge for GALC from 7.5 to 5.4 is likely to play a part in the pH dependence of this interaction.

Supplementary Figure 3

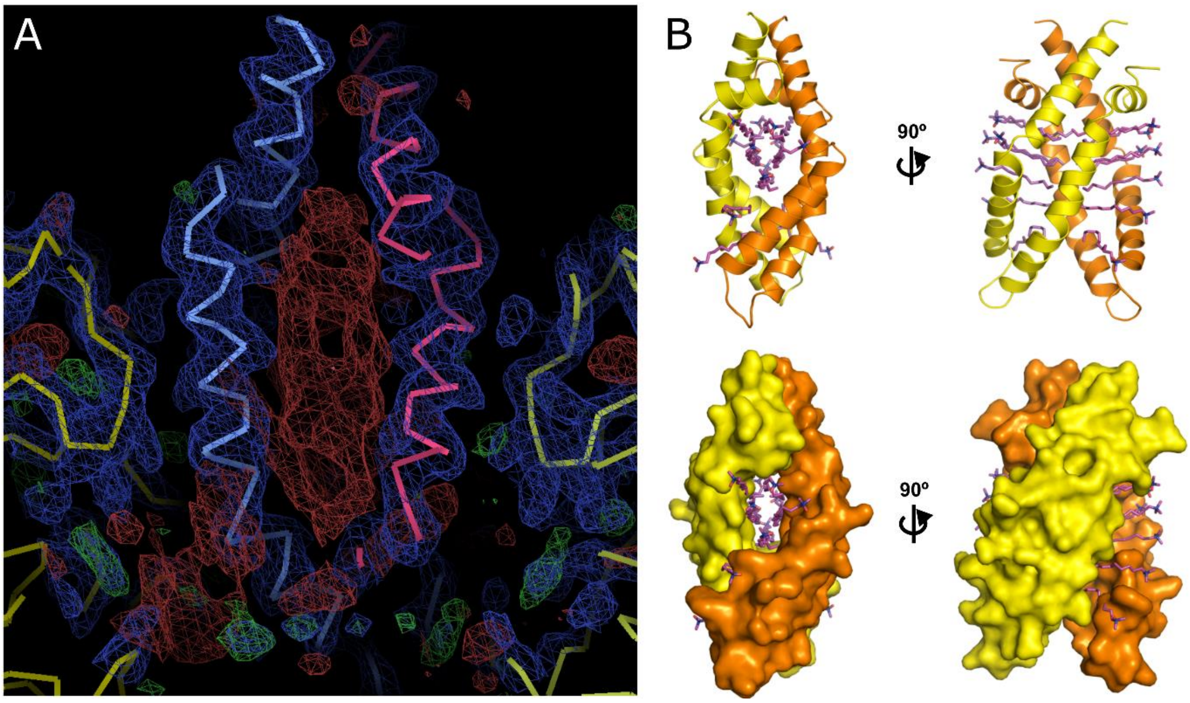

Supplementary Figure 3. Disordered LDAO detergent is present in the SapA dimer hydrophobic core. (A) The GALC-SapA backbone structure is shown as ribbons. $2F_O$-F_C (blue, 0.130 $e^-/Å^3$) and F_O-F_C (green/red for positive/negative at 3.0 σ) difference electron density maps are shown as mesh. While the solvent surrounding the molecule is devoid of features in the difference map, there is significant negative density within the cavity between SapA monomers. This is consistent with the cavity being filled with a disordered acyl chains, their average electron density being lower than that of the bulk (aqueous) solvent surrounding the GALC-SapA proteins within the crystal [2]. Phases for map calculation were obtained by autobuster refinement of the model with the setting "AnalyseVoids=no", ensuring that the cavity between SapA molecules was modelled as bulk solvent. (B) The volume of the SapA dimer cavity can accommodate 10-14 LDAO molecules. Ribbon (top) and surface (bottom) diagrams showing two orientations of the SapA dimer with 14 molecules of LDAO positioned based on the lipoprotein disc structure (PDB 4DDJ, [3]).

Supplementary Figure 4

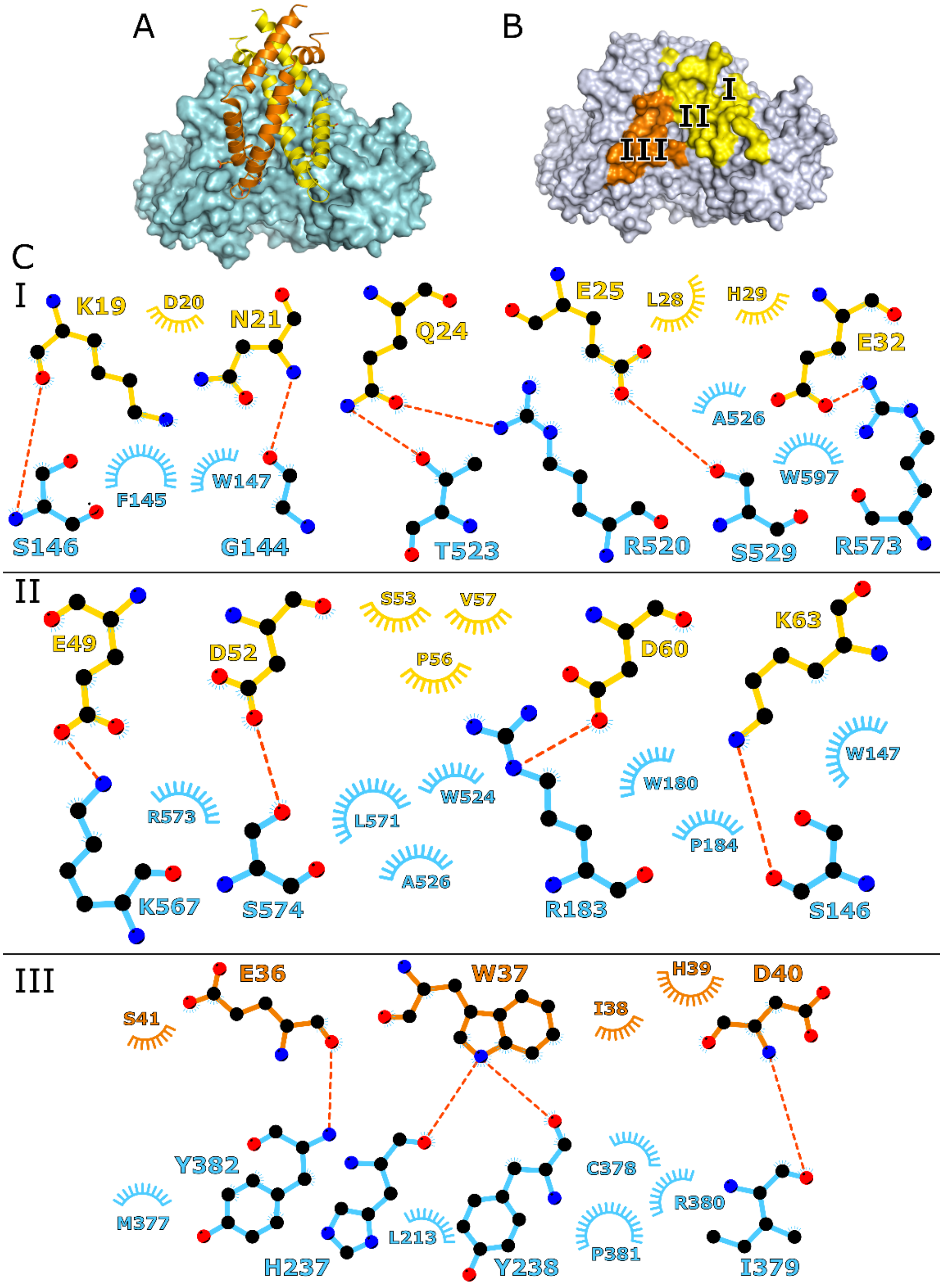

Supplementary Figure 4. Details of interactions at the GALC-SapA interface. (A) The GALC surface (cyan) is shown with ribbon diagrams of SapA chains 1 (yellow) and 2 (orange). (B) The GALC surface (grey) is shown in the same orientation as (A) with interacting regions contacted by SapA chain 1 (yellow) and chain 2 (orange) highlighted. The interacting regions are designated I, II and III, based on their interactions with SapA residues 19-32, 49-63 and 36-40, respectively, as described in Figure 4A. (C) Illustration of the protein-protein interactions between GALC (cyan) and SapA (yellow and orange for chains 1 and 2, respectively) for regions I, II and III identified in (B). Hydrogen bonds are shown as red lines, hydrophobic interactions as fanned lines, and selected atoms of interacting residues are shown (carbon, black; nitrogen, blue; oxygen, red).

Supplementary Figure 5

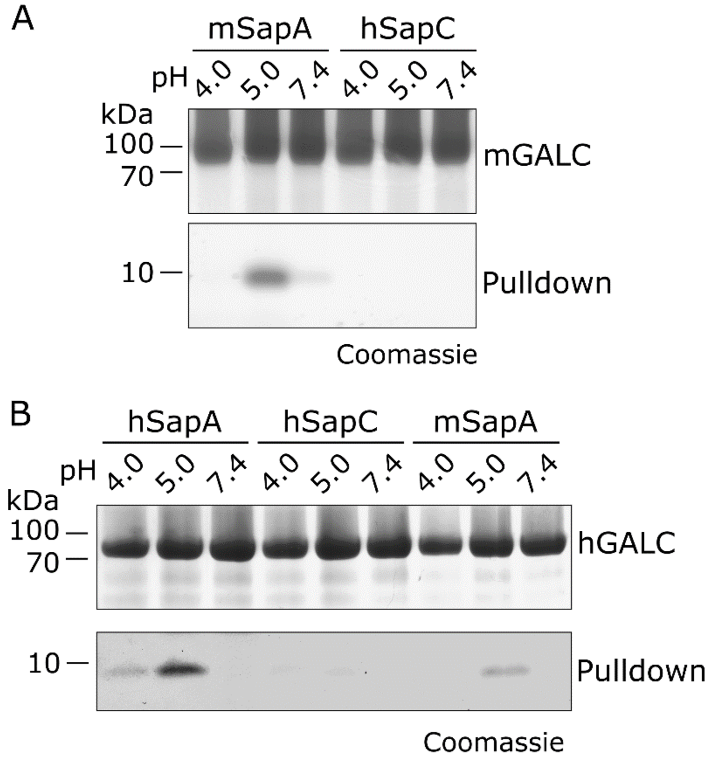

Supplementary Figure 5. GALC binding to SapA but not SapC. (A) Coomassie-stained SDS-PAGE pulldown with immobilised murine GALC (mGALC) illustrating specific binding to murine SapA (mSapA), but not human SapC (hSapC). (B) Coomassie-stained SDS-PAGE pulldown with immobilised human GALC (hGALC) illustrating binding to both human and murine SapA, but not to hSapC.

Supplementary Figure 6

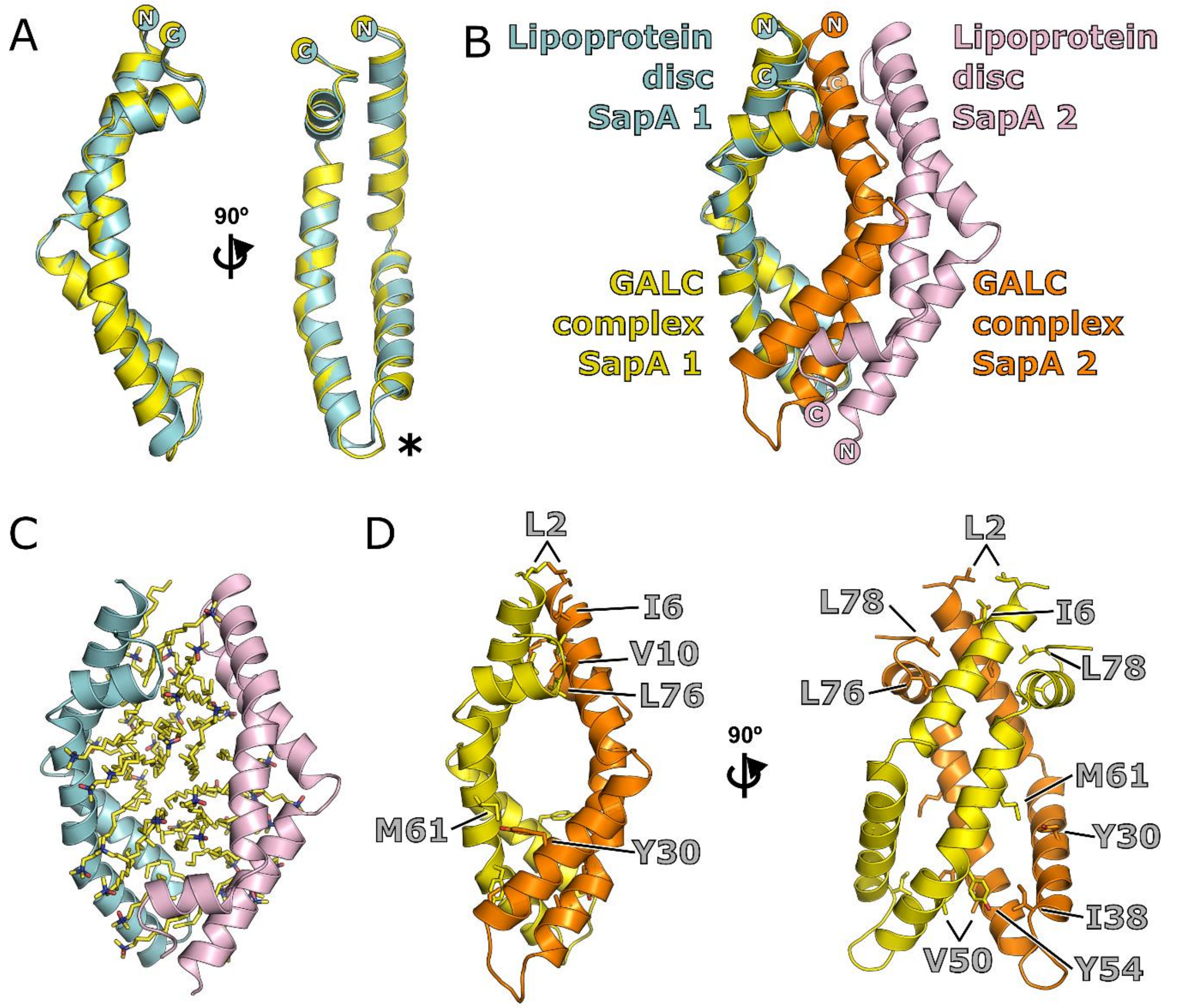

Supplementary Figure 6. Comparison of SapA dimer structures. (A) The conformation of the SapA monomers in the GALC complex (yellow) and the lipoprotein disc (cyan) are almost identical, except for a small loop movement at residues W37 and I38 (*). (B) In the dimer structures the second SapA chains (orange and pink, respectively) are in different positions and opposite orientations (N and C termini marked with circles) with the lipoprotein disc structure possessing a larger cavity. (C) The lipoprotein disc does not possess direct protein-protein interactions and is instead maintained via interactions with ordered LDAO molecules (yellow sticks). (D) The SapA dimer in complex with GALC possesses several protein-protein contacts, including multiple hydrophobic interactions where the termini come together (L2, I6, V10, L76 and L78) and contacts near the interface with GALC (Y30, I38, V50, Y54 and M61).

Supplementary Figure 7

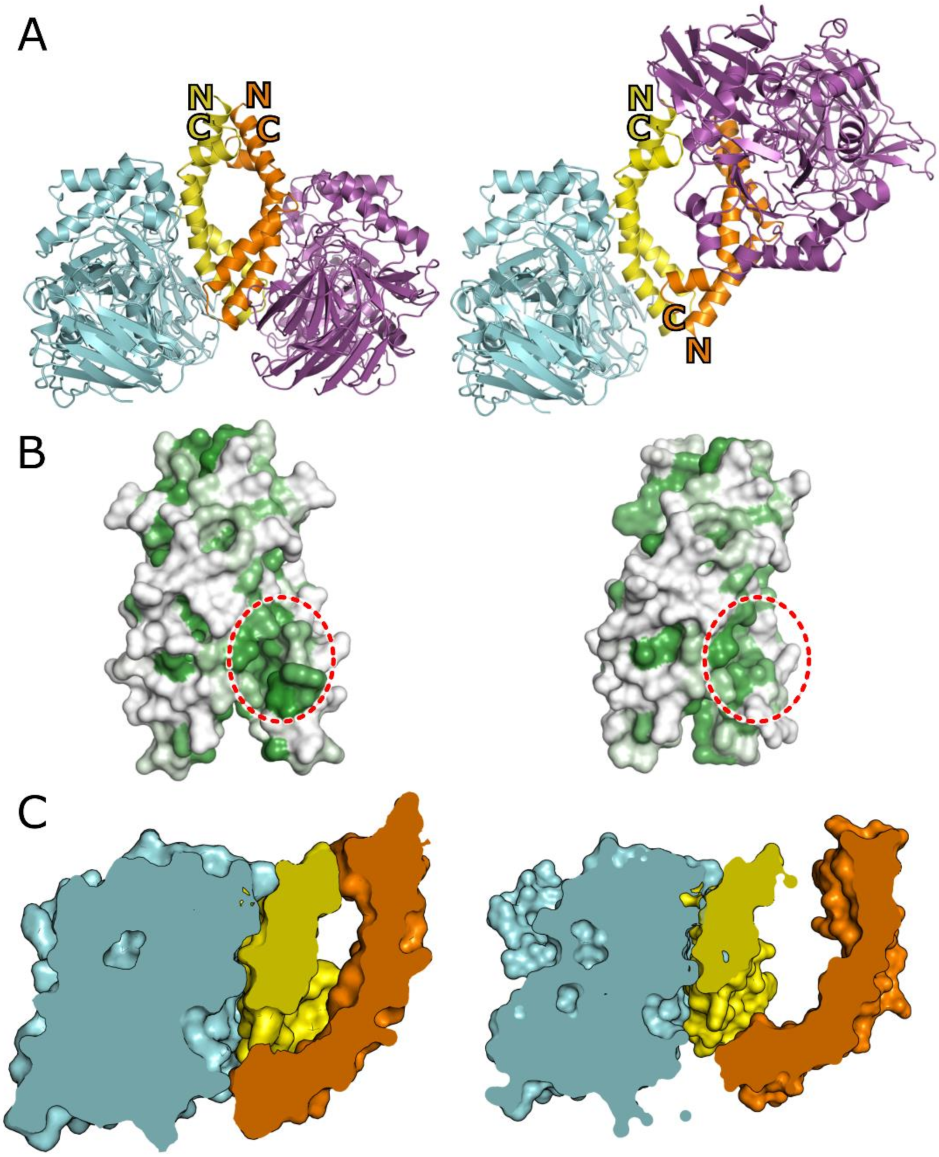

Supplementary Figure 7. Comparison of the GALC-SapA complex with an alternative SapA dimer model. Left panels are the structure determined in this study. Right panels represent a model where the SapA dimer arrangement is based on the lipoprotein disc structure (PDB ID 4DDJ, [3]). (A) Ribbon diagram illustrating the alternative orientation of the second SapA and GALC where the SapA dimer from the lipoprotein disc structure is used. Termini are labelled to show the flipped arrangement of second SapA chain. Orientation shown is as in panel C of Figure 2. (B) Residue hydrophobicity (green) is mapped onto the surface of the SapA dimers. Orientation shown is as in the central panel of Figure 3A. The region that lies over the GALC active site is circled (red dashed line). (C) Cross-section through these structures reveals differences in the channel stretching from the GALC active site into the SapA hydrophobic cavity. The channel based on the lipoprotein structure is more open than the channel in our structure and exposes the channel to more bulk solvent. Orientation shown is as in panel B of Figure 3.

Supplementary Figure 8

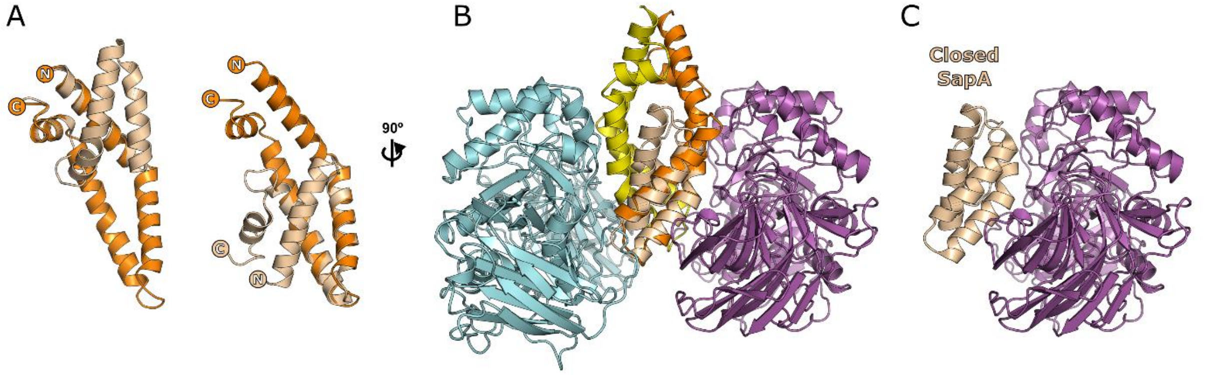

Supplementary Figure 8. Overlays of closed SapA with the open SapA in our structure. (A) Closed SapA (PDB ID 2DOB, [4]) was docked onto open SapA via superposition of the terminal helices (residues 1-20 and 65-80, left) or the central helices (residues 24-59). (B) Docking of closed SapA via the central helices of SapA within the context of the GALC-SapA complex would block formation of a heterotetramer, but would maintain some of the critical interactions identified in this study. (C) The docking of closed SapA onto a monomer of GALC buries a surface area of 669 $Å^2$ (compared with 1366 $Å^2$ for the tetramer) and is not predicted by ePISA to form a stable complex (CSS=0, [5]).

Supplementary Figure 9

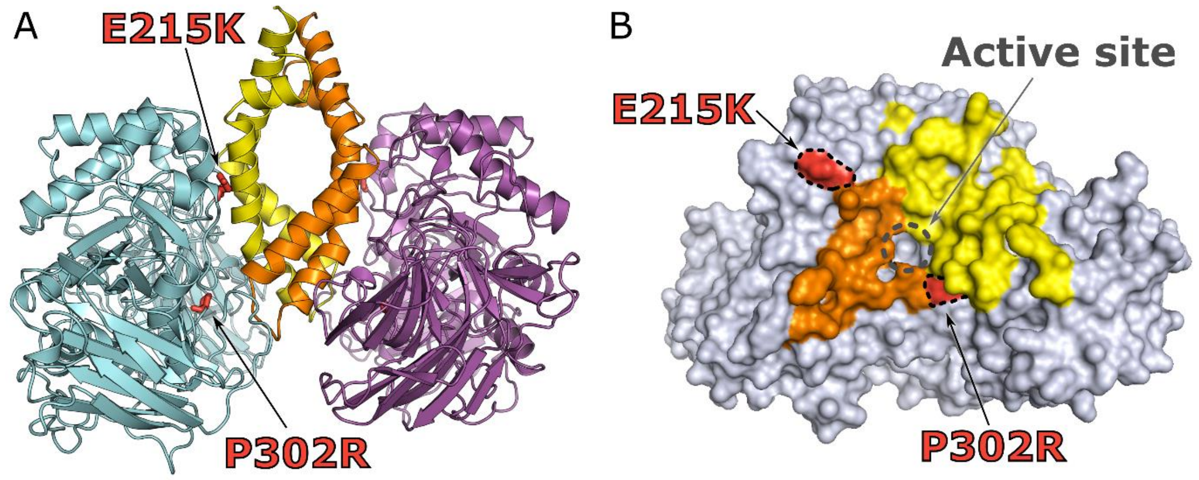

Supplementary Figure 9. Two disease-causing mutations of GALC lie near the interaction interface with SapA. (A) Ribbon diagram of the GALC-SapA complex illustrating the position of two disease-causing mutations E215K and P302R (red sticks). (B) Surface diagram illustrating the interaction surface of GALC as shown in Figure 2D highlighting the proximity of the two disease-causing mutations E215K and P302R (red surface with dashed outline) to the saposin-binding epitope.

Supplementary Figure 10

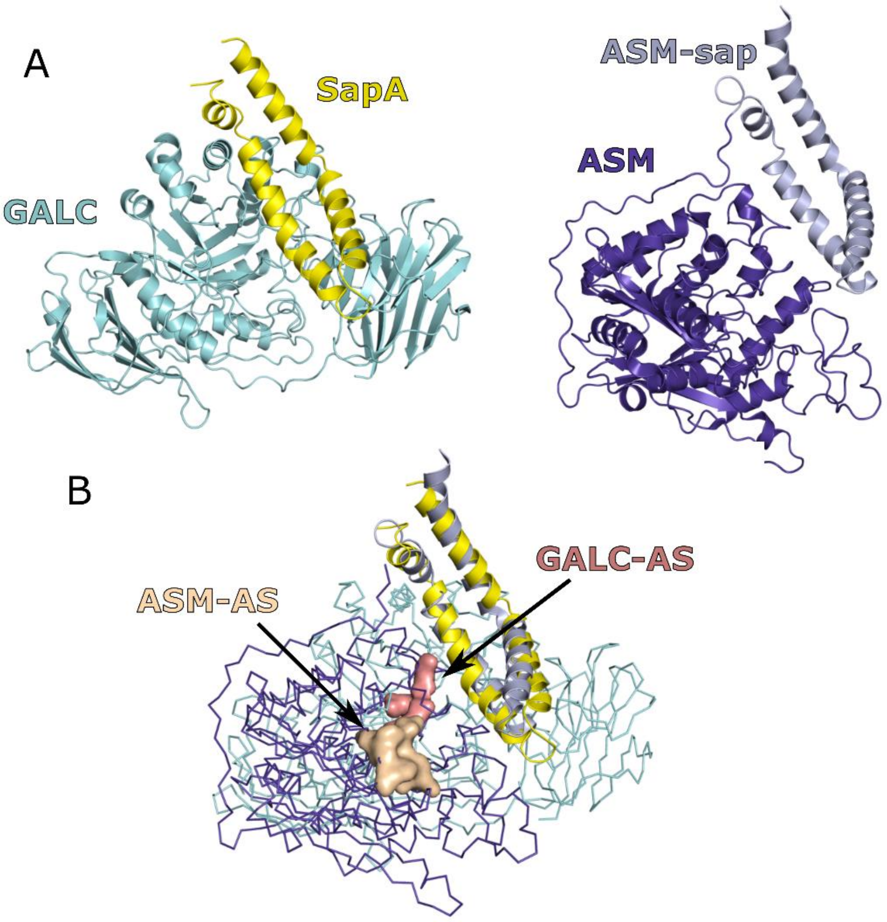

Supplementary Figure 10. Comparison of the GALC-SapA complex with acid sphingomyelinase (ASM). (A) Side-by-side structures are shown of the GALC-SapA complex (left panel) and ASM (right panel, PDB ID 5JG8, [6]) following superposition of the saposin-like domain of ASM (light purple) onto SapA (yellow). This overlay allows for the comparison of the orientations of the catalytic domain of ASM (purple) with GALC (cyan). (B) Superposition via the saposin domains (ribbons) and display of the active sites of ASM (AS, wheat surface) and GALC (AS, pink surface) reveal the similar but not identical positions of the active sites. For clarity, the catalytic domains of GALC and ASM are illustrated as backbone traces.

Supplementary Figure 11

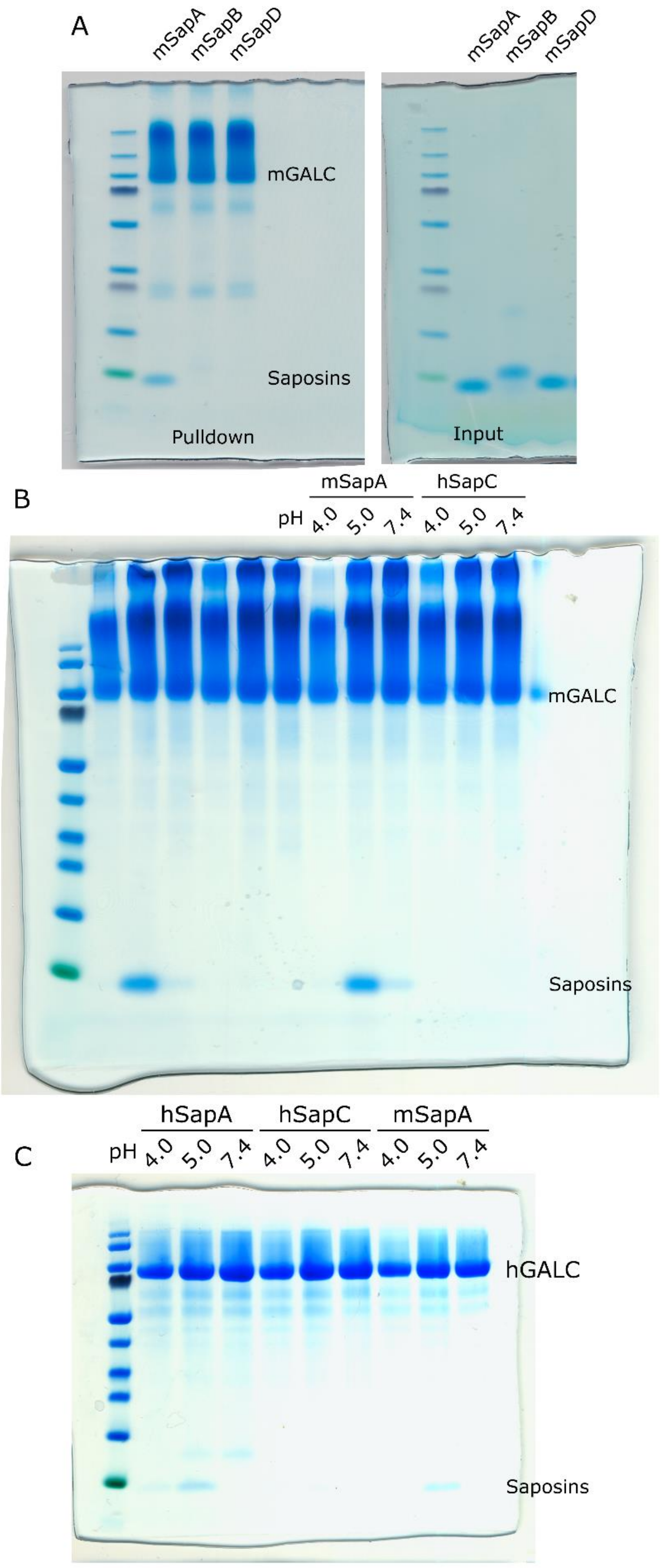

Supplementary Figure 11. Uncropped images of gels used in: (A) Figure 5C and (B and C) Supplementary Figure 5.

Supplementary Table 1. Primer sequences used in this study

Construct	Forward Primer	Reverse Primer
mSapA K19E	GAAGCAGGCAATCTGCTGGAAGATAATGCAACCCAAG	CTTGGGTTGCATTATCTTCCAGCAGATTGCCTGCTTC
mSapA N21Y	GGCAATCTGCTGAAAGATTATGCAACCCAAGAAGAAATC	GATTTCTTCTTGGGTTGCATAATCTTTCAGCAGATTGCC
mSapA Q24K	CTGCTGAAAGATAATGCAACCAAAGAAGAAATCCTGCAC	GTGCAGGATTTCTTCTTTGGTTGCATTATCTTTCAGCAG
mSapA E25K	GAAAGATAATGCAACCCAAAAAGAAATCCTGCACTATCTGG	CCAGATAGTGCAGGATTTCTTTTTGGGTTGCATTATCTTTC
mSapA L28R	CAACCCAAGAAGAAATCCGGCACTATCTGGAAAAAAC	GTTTTTTCCAGATAGTGCCGGATTTCTTCTTGGGTTG
mSapA L28N	CCAAGAAGAAATCAATCACTATCTGGAAAAAAACC	GGTTTTTTCCAGATAGTGATTGATTTCTTCTTGG
mSapA E32R	GAAATCCTGCACTATCTGAGAAAAAACCTGCGAATGGATTC	GAATCCATTCGCAGGTTTTTTCTCAGATAGTGCAGGATTTC
mSapA W37D	CCTGCGAAGATATTCATGATAGCAGCC	GGCTGCTATCATGAATATCTTCGCAGG
mSapA W37F	CCTGCGAATTCATTCATGATAGCAGCC	GGCTGCTATCATGAATGAATTCGCAGG
mSapA W37S	CCTGCGAATCGATTCATGATAGCAGCC	GGCTGCTATCATGAATCGATTCGCAGG
mSapA E49K	AGCGCAAGCTGTAAAAAAGTTGTTGATAGCTAT	ATAGCTATCAACAACTTTTTTACAGCTTGCGCT
mSapA D52K	TGTAAAGAAGTTGTTAAAAGCTATCTGCCGGTG	CACCGGCAGATAGCTTTTAACAACTTCTTTACA
mSapA D60K	CTGCCGGTGATTCTGAAAATGATTAAAGGTGAA	TTCACCTTTAATCATTTTCAGAATCACCGGCAG
mSapA K63E	ATTCTGGATATGATTGAAGGTGAAATGAGCAAT	ATTGCTCATTTCACCTTCAATCATATCCAGAAT
mSapA E65K	GATATGATTAAAGGTAAAATGAGCAATCCGGGT	ACCCGGATTGCTCATTTTACCTTTAATCATATC
hGALC	TCTGATATCCATCATCATCATCATCATATCGAGGGCCGCGGCAGCTACGT GCTCGACGACTCCGACGGGC	CCAAGGATCCGAATTCTTAGCGTGTGGCTTCCACAAGAAAGTTGTCA

Supplementary References

1 Hill, C. H., Graham, S. C., Read, R. J. & Deane, J. E. Structural snapshots illustrate the catalytic cycle of beta-galactocerebrosidase, the defective enzyme in Krabbe disease. *Proc Natl Acad Sci U S A* **110**, 20479-20484, (2013).

2 Cockburn, J. J. *et al.* Membrane structure and interactions with protein and DNA in bacteriophage PRD1. *Nature* **432**, 122-125, (2004).

3 Popovic, K., Holyoake, J., Pomes, R. & Prive, G. G. Structure of saposin A lipoprotein discs. *Proc Natl Acad Sci U S A* **109**, 2908-2912, (2012).

4 Ahn, V. E., Leyko, P., Alattia, J. R., Chen, L. & Prive, G. G. Crystal structures of saposins A and C. *Protein Sci* **15**, 1849-1857, (2006).

5 Krissinel, E. & Henrick, K. Inference of macromolecular assemblies from crystalline state. *J Mol Biol* **372**, 774-797, (2007).

6 Xiong, Z. J., Huang, J., Poda, G., Pomes, R. & Prive, G. G. Structure of Human Acid Sphingomyelinase Reveals the Role of the Saposin Domain in Activating Substrate Hydrolysis. *J Mol Biol* **428**, 3026-3042, (2016).
